# How to build up big team science: a practical guide for large-scale collaborations

**DOI:** 10.1098/rsos.230235

**Published:** 2023-06-07

**Authors:** Heidi A. Baumgartner, Nicolás Alessandroni, Krista Byers-Heinlein, Michael C. Frank, J. Kiley Hamlin, Melanie Soderstrom, Jan G. Voelkel, Robb Willer, Francis Yuen, Nicholas A. Coles

**Affiliations:** ^1^ Center for the Study of Language and Information, Stanford University, Stanford, CA, USA; ^2^ Department of Psychology, Stanford University, Stanford, CA, USA; ^3^ Department of Sociology, Stanford University, Stanford, CA, USA; ^4^ Department of Psychology, Concordia University, Montreal, Quebec, Canada; ^5^ Department of Psychology, University of British Columbia, Vancouver, British Columbia, Canada; ^6^ Department of Psychology, University of Manitoba, Winnipeg, Manitoba, Canada

**Keywords:** big team science, science of team science, meta-science, collaboration

## Abstract

The past decade has witnessed a proliferation of big team science (BTS), endeavours where a comparatively large number of researchers pool their intellectual and/or material resources in pursuit of a common goal. Despite this burgeoning interest, there exists little guidance on how to create, manage and participate in these collaborations. In this paper, we integrate insights from a multi-disciplinary set of BTS initiatives to provide a how-to guide for BTS. We first discuss initial considerations for launching a BTS project, such as building the team, identifying leadership, governance, tools and open science approaches. We then turn to issues related to running and completing a BTS project, such as study design, ethical approvals and issues related to data collection, management and analysis. Finally, we address topics that present special challenges for BTS, including authorship decisions, collaborative writing and team decision-making.

## 1. Introduction

Over the past decade, social and behavioural scientists have increasingly engaged in *big team science* (BTS): collaborations wherein a comparatively large number of researchers pool their intellectual and/or material resources to pursue a common goal [1].^1^ For example, researchers have recruited large teams to help design studies, collect large datasets, examine different ways of analysing data, write papers and so on. This ultra-collaborative approach to science has afforded many benefits, including increases in (i) the power to study complex research questions, (ii) the diversity (e.g. epistemic, geographical, cultural) of the perspectives that lead and/or are captured in research,^2^ and (iii) the sharing of expertise and best practices (for more details, see [3,4]). Despite these benefits, there is little guidance on how to create, manage and participate in these collaborations. The purpose of this paper is to fill that gap.

Our practical guide to BTS is designed to be used by anyone—particularly because our experience shows that such initiatives *can* be created and managed by individuals from a wide variety of backgrounds and levels of training. Although BTS initiatives have historically involved large budgets managed by well-established institutions (e.g. the European Organization for Nuclear Research [5], the Human Genome Project [6] and the Adolescent Brain Cognitive Development Study [7]), an increasing number are being operated in a relatively grassroots manner with little-to-no funding. Regardless of how one wishes to structure their BTS initiative, they are likely to face a host of challenges, including building a team, identifying leadership, establishing and navigating decision-making structures, dealing with issues regarding authorship and the attribution of credit, overcoming language and time zone barriers, fairly distributing workload, navigating status and power differences, and so on. In this paper, we offer a how-to guide for tackling these problems, so that researchers can maximize the benefits and avoid the potential pitfalls of BTS.

This guide is based on our own experiences (both positive and negative) creating, managing and having conversations with others who lead large BTS initiatives in the social and behavioural sciences. We primarily represent teams studying developmental psychology (*ManyBabies*), adult social and cognitive psychology (*Psychological Science Accelerator*) and social and political science (*Strengthening Democracy Challenge*). Nonetheless, we discuss similar initiatives throughout the manuscript (e.g. see electronic supplementary material for a case study on the *Many Smiles Collaboration* [8]). Although our perspectives are certainly shaped by our disciplinary backgrounds, we anticipate that many of our experiences and recommendations will be relevant for BTS initiatives across the sciences.

## 2. Before you start

### 2.1. Survey the space

Before you begin a BTS project, it is useful to first consider if a similar initiative already exists. This point may seem obvious, but it can be easy to overlook when caught up in the enthusiasm of a new idea. If an initiative similar to the one you are envisioning already exists, this can be a fantastic opportunity for you to join and contribute to an existing collaboration.

If no similar initiative exists, the next thing to consider is whether you expect there to be sufficient community buy-in to support your vision of a large-scale collaborative project. BTS projects often rely on the motivation of a critical mass of researchers to contribute their (probably scarce) resources—including time, money, infrastructure and participants. Determining whether there is sufficient motivation to contribute those resources often involves a combination of informal and formal actions. For example, many of our projects started through organic discussions between researchers at conferences, on listservs and/or on social media. However, some have found it helpful to start with a more intentional process, such as sending targeted polls and queries to listserv members.

If there is enough interest from others to support your vision, the next thing to consider is whether your idea for a BTS project is feasible. To assess feasibility, it is helpful to identify concrete examples of the types of topics you would like to address. While it can be tempting to jump right in with a complex topic, many initiatives have found it useful to start simple. For example, groups like the *Psychological Science Accelerator*, *ManyPrimates*, *ManyBirds* and *ManyDogs* focused their first projects on ideas that were relatively easy to test across a wide variety of sites and/or taxa [9–12]. Similarly, as a proof-of-concept, *ManyBabies* began with a study of a relatively non-controversial topic for which there was already strong empirical support: infants' preference for infant-directed speech [13].

### 2.2. Determining where to leverage the power of big teams

**Figure 1.**
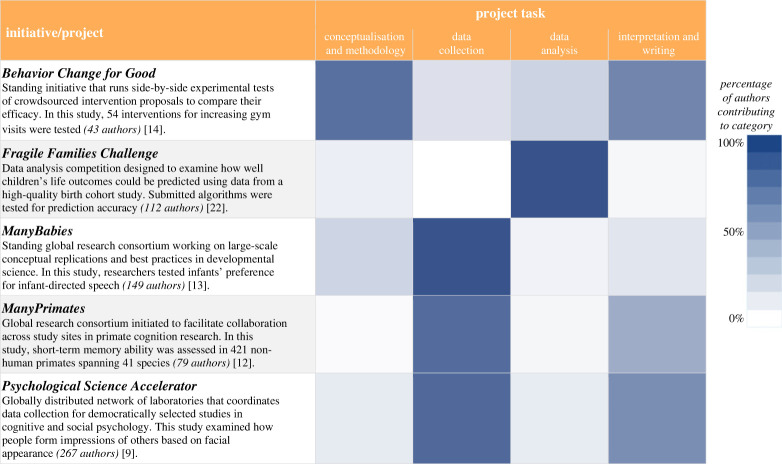
Selective BTS initiatives (arranged alphabetically). For each initiative, we approximated the percentage of authors who contributed to each aspect of the project by having two researchers (H.A.B. and N.A.C.) code the author contributions for each paper. For each project, darker shades indicate that a relatively high percentage of authors reported contributing to each aspect of the project. Citations in figure refer to [9,12–14,22]. Coding information used to generate this figure is available at https://osf.io/2p4ct/.

As you are surveying the space, it is often useful to determine where and how to leverage the power of BTS. Many BTS projects are built around the basic premise of scaling up data collection, but all phases of the research process can potentially benefit from the unique strengths that big teams offer (table 1). For example, big teams have been leveraged to (i) identify and develop research questions, hypotheses, study designs and/or materials, (ii) collect and/or process data, (iii) analyse data, and/or (iv) develop a manuscript. BTS projects differ in when and how they leverage the power of teams; many do so in multiple parts of the research process (e.g. *Psychological Science Accelerator*, *ManyBabies*), whereas others focus on just one (e.g. *Fragile Families Challenge*; figure 1). We elaborate with examples of each of these approaches below.

**Table 1.** Benefits of scaling up research components using BTS.

component of the research process	benefits	examples
conceptualization and methodology	leverage ‘wisdom of the crowd' to discover new questions	Behaviour Change for Good [14], Beck *et al*. [15], Strengthening Democracy Challenge [16], Many Smiles Collaboration [8], Landy *et al*. [17]
	increase diversity/representation of research questions	
	identify potential confounds early in process	
	consider methodological appropriateness for various cultures/regions	
	develop consensus best test by involving people from diverse theoretical ‘camps’	
data collection	increase sample size (increased power to detect effects; possible to test additional sources of heterogeneity)	Psychological Science Accelerator [9], ManyBirds [10], ManyDogs [11], ManyPrimates [12], ManyBabies [13]
	increase diversity of sample to allow for improved inferences about generalizability	
data analysis	pool expertise to determine state-of-the-art analysis techniques	Botvinik-Nezer *et al*. [18], Silberzahn *et al*. [19]
	examine how analysis choices affect results and interpretations of data	
interpretation and writing	multiple perspectives can lead to novel insights	Open Science Collaboration [20], Lakens *et al*. [21]
	individuals are forced to strengthen justifications or reconsider preconceptions	
	distribution of labour	

Some BTS projects are created to crowdsource multiple hypotheses or interventions to address a research question [14–16,23–26]. Doing so often allows these projects to draw from multiple sources of expertise. For example, the *Strengthening Democracy Challenge* [16] invited researchers from across the social sciences, practitioners working in non-profit organizations and activist groups to submit ideas for interventions hypothesized to improve Americans' commitment to democratic principles. Academic and non-academic teams submitted 252 interventions, out of which 25 were selected in consultation with an advisory board. Those 25 interventions were then tested in a large-scale (approx. 32 000 participants) online survey experiment. Such projects thus leverage the wisdom-of-the-crowd to generate a broad set of ideas, which can then be systematically compared through a large standardized data collection effort. This is particularly important given that many societal issues—such as threatened democratic structures, global pandemics and climate change—have time-sensitive needs for data-driven insights.

Similarly, large teams can be beneficial when it comes to the design and development of procedures and materials. For example, despite spending nearly a year designing a study with a smaller group of experts, feedback from a broader set of collaborators on the early design for the *Many Smiles Collaboration* led to substantial refinements [8]. As another example, the second project run by *ManyBabies* brought together researchers with competing theoretical orientations to design a maximally informative test of outstanding debates in developmental psychology about whether or not infants possess a representational theory of mind [27]. Explicitly crowdsourcing multiple study designs can also allow teams to examine methodological choices as a source of inferential heterogeneity. For example, Landy *et al*. [17] examined how methodological choices impact research results by inviting 15 research groups to design studies to test a given research question.

Scaling up data collection via BTS allows distributed teams composed of multiple laboratories to generate much larger sample sizes than individual laboratories can (e.g. [9,13,20,28]). Larger samples, of course, often afford greater statistical power, which is particularly important when studying small effects, testing complex interactions or running analyses that need corrections for multiple comparisons. Importantly, the resulting samples are often not only bigger but also more diverse and representative of populations outside the heavily studied regions of North America and Western Europe. Increased sample diversity allows teams to (i) make more informed inferences about the generalizability of observed phenomena and also (ii) examine sample-based sources of heterogeneity (e.g. culture, methodology). For example, the first *ManyBabies* project tested infants around the world using three procedures (head-turn preference, central fixation and eye tracking) and compared dependent variable effect sizes by participant native language and procedure [13].

BTS groups with large analysis teams can benefit in many of the same ways mentioned above with regard to study design. Teams of analysts can pool expertise to determine state-of-the-art approaches (e.g. in shared task projects [22]), probe the robustness of statistical conclusions [21], identify potential issues and examine data processing/modelling choices as a source of inferential heterogeneity [18,19]. Multiple analysts can also compete to produce algorithms that can predict outcomes based on training data, such as in the *Fragile Families Challenge* [22]. In this project, competitors were given a set of training data containing predictor variables and life outcomes for a subset of children and tasked with designing an algorithm to predict outcomes for a held-out set of test data.

Finally, large teams can also be leveraged at the end of the research process, during results interpretation and manuscript writing. As in the other phases, pooled expertise across diverse stakeholders can lead to novel insights, and disagreements at this stage can force contributors to strengthen their justifications or reconsider preconceptions (e.g. [21]). Of course, a division of labour can lead to lighter work for all involved (for a guide to writing collaborative manuscripts, see [29]).

When considering when and how to scale up your project, it is important to keep in mind that different parts of the research pipeline require different organizational structures as they grow. For example, projects that crowdsource hypotheses will require more organization and contributors early in the process, whereas projects that crowdsource data collection may not recruit the bulk of their partners until after the research questions are fully developed. In some cases, available resources and interest may factor into which elements are feasible to crowdsource or scale up.

## 3. Starting the project

By the time you have decided to form a BTS project, identified a preliminary topic or focus, decided what needs to be ‘big' and confirmed interest in collaboration, you have reached an important milestone: you are a leader of a BTS project! Push through any feelings of impostor syndrome; the only required qualification for leading a BTS effort is doing it. We have found that others in the field are often grateful—rather than judgemental—that someone else is making the effort to get a BTS project off the ground. If you have not already done so, it is often useful to next think about how to structure the collaboration. How will you build the full team? Will there be formal leadership? Will there be formal governance? What tools will be used? And to what extent will the outputs be made openly available?

In our experience, most BTS initiatives eventually grapple with these questions. What varies, however, is *when* they do so. Some initiatives opt to make these decisions from the onset—especially when these teams seek support from funders who expect well-established plans. Doing so, however, can require a substantial investment of time and energy at the beginning stages of a project. This can potentially overwhelm the team before they even get the chance to dive into the more exciting parts of BTS, such as designing, conducting and evaluating the results of a study. Thus, other initiatives—particularly those that are more grassroots—tend to tackle these questions as they go, opting to ‘build the ship as they sail it'. The major drawback of this approach is that it can lead to mismatched expectations, role uncertainty and procedural mistakes (e.g. mismatched data files). We recommend, when appropriate, a hybrid approach: establishing a minimal set of standards at the onset of the collaboration and elaborating upon these standards as the initiative matures.

### 3.1. Building the team

BTS, of course, requires building a team. But who gets the opportunity to join that team? Conventionally, participating in BTS initiatives has been an invite-only affair. The benefit of such an approach is that it can help ensure that collaborators share similar values, meet particular expectations for best practices, work well together and trust one another. This approach also conforms with traditional funding models, which require members of a team to be identified as part of a project proposal. The drawback is that such an arrangement often conflicts with the drive to make science more diverse and inclusive. Many groups—like *ManyBabies*, the *Psychological Science Accelerator* and *ManyPrimates*—have thus embraced an open collaborative model: anybody is eligible to participate regardless of their discipline, career stage, institutional affiliation and so on. Such an approach requires researchers to grapple with differences in values, norms and expectations. However, it provides extremely fertile grounds for considering, transferring and integrating diverse perspectives in science.

Regardless of whether a BTS initiative is open or closed, it is helpful to consider the value proposition for potential collaborators: why should they join your BTS initiative? Successfully recruiting a BTS team essentially means convincing a large number of people that their expected gains outweigh their expected costs. Most BTS initiatives will require researchers to invest time and/or resources. In return, collaborators may receive co-authorship on a publication, exclusive or early access to a dataset, payment, training opportunities, networking opportunities and/or the satisfaction of contributing to what is often a high-impact scientific endeavour. For example, the first *ManyBabies* project offered contributors inclusion in consortium authorship on a large publication in exchange for collecting data from at least 16 infants via an established procedure. For the *Strengthening Democracy Challenge*, competing to design the most effective intervention provided several benefits, including knowledge that one is helping address an important societal problem, free testing of ideas, co-authorship and prize money. To reduce risk to contributors, many initiatives have found it helpful to pursue Registered Reports, which are peer-reviewed and accepted in-principle *before* data collection commences [30]. Whatever the specific balance of costs, benefits and risk may be, communicating this balance helps establish clear expectations with potential collaborators.

Once you have worked out why people should join your team, where should you go to find potential collaborators? Recruiting BTS collaborators involves a process that is somewhat foreign to many academics: constructing an outreach strategy beyond their immediate networks. We are not aware of any investigations of the relative efficacy of different recruitment strategies, so we (and others) have often used as many strategies as possible: social media posts, blog posts, emails to professional listservs and ‘cold-call' emails. In addition, coordinating with researchers who have large collaborative networks can be helpful in generating a list of potential collaborators and sharing calls for participation. BTS initiatives that seek to improve diversity and inclusion often have to supplement these approaches with targeted outreach efforts.

As part of spreading the word, it can be helpful to implement a simple point of entry for further participation, for example a simple Project Interest form. These forms can reduce the barrier to joining, particularly for early career researchers and/or those from under-represented groups, institutions or locations who might be hesitant to contact project organizers directly. It can also help in gathering systematic information about collaborators, such as their academic position, affiliation and skillset. It can also help to ensure that a comprehensive list of collaborators and their contact information is stored in a single place for the duration of the study—a key concern as the number of collaborators and project timeline grow. For more discussion of how to conduct outreach and onboard new members, see [31].

### 3.2. Choosing a leadership team

BTS initiatives vary in their organizational structures and decision-making processes. We begin by distinguishing between network-level and project-level leadership. For one-off BTS projects (or those that are unsure of long-term plans beyond a first project), project leaders—with the support of team members—often can handle the initial demands of BTS leadership outlined here. For BTS initiatives that are intended to be standing networks with multiple projects, it is useful to have a small centralized governing board that is responsible for project oversight and governance. For *ManyBabies*, a group of seven individuals came together to form a governing board at the time of group formation in 2016, and two additional members were added via an election in 2022. In addition to guiding the overall initiative, these governing board members often also serve in leadership roles on specific projects.

In our experience, almost all successful BTS projects have a core leadership group (e.g. two to five people) that (i) takes responsibility for guiding the project and (ii) commits to a significant investment of effort into the project to ensure it moves forward. In addition, many have found it helpful to identify a slightly larger supporting team that helps the leadership group by taking responsibility for specific aspects of the project, such as methodological planning, analyses and writing. In our experience, establishing clear expectations about who is leading what aspects of a project helps to avoid many of the potential pitfalls of collective behaviour, including diffusion of responsibility, role confusion, uncertainty regarding who to contact for specific problems and ambiguity about who to credit for particular contributions. Furthermore, because the organizational overhead of BTS is unusually large, having many people assist in these leadership and supporting roles is often the only way of accomplishing the goals of the BTS initiative. For detailed discussions of how two BTS groups, *ManyBabies* and the *Psychological Science Accelerator*, formed their network-level (Governing Board) and project-level leadership teams, see [32,33].

Many BTS initiatives benefit from a diverse leadership team. Often, this means recruiting a leadership team that is diverse in terms of ethnicity, location, career stage, professional background and/or other relevant dimensions (see [34–37] for more information on recruiting and supporting diverse teams in science). In addition to its moral justification, diversity in BTS leadership often confers epistemic and practical benefits. For example, having a culturally diverse leadership team in multi-national research brings varied perspectives on both methodological and theoretical issues, which may lead to more robust scientific insights. Furthermore, this leadership structure helps the team identify scenarios where certain methodologies and measurement strategies are inappropriate or likely to lead to artefacts that will thwart efforts to make meaningful cross-cultural comparisons and/or lead to practical methodological barriers across different contexts of research. Diversity in leadership also helps the team understand the potential barriers that other researchers with similar backgrounds may face when participating in BTS.

Diverse leadership can also bring its own set of complexities, and each dimension of diversity presents unique sets of challenges. As such, we limit our discussion here to two examples. The first example concerns geographical diversity in leadership: scheduling across time zones. There is simply no time that works well for everybody around the world, and people are unlikely to join BTS leadership if meetings are consistently scheduled at inconvenient times, such as the middle of the night or during family dinners. We have thus found it useful to visualize meeting times across time zones using freely available tools like ‘World Time Buddy' [38]. When there is no time that is convenient for all, there are several strategies that can be effective. This includes (i) rotating meeting times to accommodate different time zones, so that the expectation to meet at inconvenient times (or necessity to miss the meeting entirely) is shared more equitably, (ii) recording meetings for those who cannot be accommodated, (iii) scheduling multiple meeting times to discuss the same topic, and (iv) creating workflows that reduce the frequency of meetings and allow people who cannot attend meetings to still provide updates and weigh in on key decisions.

The second example concerns career stage diversity in leadership, which requires navigating power structures. For example, graduate and postdoctoral researchers bring enthusiasm, new perspectives and organizational and technical skills. However, when dealing with senior co-leaders, they may find that existing power structures get in the way of their ideas being heard, their directives being followed, and their dissenting opinions being considered. It is crucial that more senior members of team leadership be aware of these power dynamics and take action to create an inclusive collaborative atmosphere. For example, this may involve (i) ensuring that all members of leadership get time allocated to discuss their perspective on an issue, (ii) using anonymous voting, and (iii) offering support and guidance when early career researchers encounter issues. This guidance also helps when considering other aspects of diversity, such as gender and race/ethnicity, topics which have been covered extensively beyond BTS contexts (e.g. [39,40]).

### 3.3. Creating governance

Although you might not think that your group needs governance documents, we have seen BTS initiatives significantly disrupted by conflicts arising from differing expectations about timelines, how to handle disagreements and disputes over ownership of research products (e.g. publications and data). Just like any collaboration, it is important in BTS that team members understand and agree upon the nature and goals of the collaboration. To facilitate this process, we have found that BTS projects benefit from the onset from a few basic and easily implementable governance documents, including collaboration agreements, codes of conduct and forms for contribution tracking (each of which is described below). BTS is a large investment, and these documents help ensure its investors understand its terms. Such documents do not need to be long, complex or written in formal legalese. The key goal of these documents is ensuring that all contributors understand and agree to a basic plan: what their role is in the project, what they are committing to doing and not doing and what they are being promised in return. Examples of these documents are often available on the websites of existing BTS collaborations or upon request, and they can be reworked to meet the needs of your initiative. For example, *ManyBabies* uses collaboration agreements adapted from a template provided by the *Psychological Science Accelerator*; see [41] for shared resources.

#### 3.3.1. Collaboration agreements

The underlying principle of a collaboration agreement is to provide every contributor with clarity and transparency about the project's goals, expectations and policies [42]. At a minimum, these agreements should include (i) definitions of contributor roles and responsibilities, (ii) criteria for authorship and planned authorship model (e.g. consortium authorship versus individual names; information about authorship ordering), (iii) information about how decisions will be made, how conflicts will be resolved and how changes to the agreement will be handled (e.g. by group consensus, by majority vote or by project leaders), and (iv) the expected outcomes/deliverables of the project (e.g. publication(s), datasets).

We recognize that some researchers may find the notion of a written collaboration agreement off-putting, particularly within more grassroots BTS endeavours. One way to mitigate this impression is to emphasize what an agreement adds to a project (e.g. transparency, accessibility) rather than what it can prevent (e.g. conflict, authorship disputes) and to create short, separate policies for key issues (e.g. authorship and publication). Many of us believe that every collaboration—big or small—stands to benefit from the clarity provided by collaboration agreements. Much like insurance, it is better to have an agreement and not need it than to realize you need it and not have it, by which time it is already too late.

#### 3.3.2. Codes of conduct

Many collaboration agreements clarify expectations for the structure of a project—but not for the overall conduct of the collaborators within a network or initiative. For instance, a collaboration agreement may specify that disagreements are resolved by majority vote but not say much about expectations of respect and openness while discussing those disagreements. For this reason, many BTS initiatives have instituted codes of conduct. For example, many of our groups have adopted or adapted the Contributor Covenant Code of Conduct for this purpose [43]. For BTS projects which do not anticipate the need for a standalone code of conduct (e.g. ‘Many Analyst' projects with minimal direct interactions between collaborators), expectations regarding respectful behaviour and communication can be included as a supplement to the collaboration agreement. A nice summary of resources related to BTS codes of conduct can be found at [31].

### 3.4. Identifying tools for collaboration

Like any aspect of human activity, the success and efficiency of BTS initiatives is partly based on the tools they use. Tools can help—or hinder—efficiency, organization and engagement. For example, co-editing a manuscript through an online browser-based tool is more *efficient* than having collaborators pass around dozens of edited documents through email. Having a single cloud-based shared folder for storing documents like ethics approvals is more *organized* than having these files spread out across collaborators' local computers. Creating an easy-to-locate shared calendar with links to virtual meeting rooms and feedback documents better facilitates *engagement* than passing this information along informally.

Table 2 shows a list of recommended tools for communication, collaboration and file storage—along with notes on what we view as their pros and cons.^3^ As you evaluate which tools to use, it is important to consider both the usefulness and likelihood that members of the team will be willing and able to use them. Indeed, even the most powerful tool will ultimately be of limited value if only a small fraction of the team actually uses it. Of course, sometimes more complex or unfamiliar tools will be deemed necessary for the success of a project. In these cases, you can consider providing easy-to-access training on the tool for interested team members. For further guidance on collaboration tools and practices, see [44].

**Table 2.** A selection of tools for collaboration, their pros, cons and alternatives.

collaborative need	example tool^a^ (alternatives)	pros	cons
writing (documentation, manuscripts)	Google Docs (Microsoft Word Online, Draft, Etherpad)	free; widely used; allows concurrent editing; can flexibly adjust permissions; allows version control; can be integrated with some third-party tools	not available in all world regions; doesn't integrate with some popular writing tools (e.g. reference managers); missing useful features (e.g. upvoting or downvoting)
file storage	Google Drive (Dropbox, Box)	free; widely used; wide familiarity and ease of use; some institution-linked drives have no limit on storage	files can be easily deleted or overwritten when access is not restricted; storage limits differ by account type (e.g. linked to an institution versus not); access issues can arise, for example, if the owner of a drive changes institutions
resource archiving and sharing	Open Science Framework (Zenodo)	free service for long-term file storage; easily connects with Google Drive and Github; allows for time-stamped pre-registration	limits on storage per project/components (currently 5 GB for private, 50 GB for public); not as intuitive to use as Google Drive
code/analysis management and storage	GitHub (GitLab)	distributed version control is crucial for effective collaboration on data analysis pipelines; good documentation; widely used; can be easily linked with the Open Science Framework	steep learning curve; requires some technical know-how and comfort; may deter some contributors
group communication *(email)*	mailman listservs (Google Groups, Mailchimp)	wide familiarity and access; effective for periodic one-way updates and/or to alert large groups of people to upcoming events or major decision points	contributors might ignore emails if they are too frequent; searching and threading can get onerous with large teams
group communication *(messaging)*	Slack (Discord)	well suited for brief exchanges involving large groups of people and/or where keeping a historical record is helpful; team members can get and provide quick responses to enquiries	hesitancy of group members to add new tools to their workflow (in addition to email and text); only useful if enough people use it; free version currently only allows access to previous 90 days
synchronous meetings *(video-conferencing)*	Zoom (Google Meet)	can allow for more nuanced communication than text; good for smaller leadership meetings but can also function as an important opportunity to create buy-in from larger team, communicate complex aspects of research protocols or create forum for consensus-based decision-making	Scheduling can be complicated for large, distributed teams; requires certain Internet speed
project management	Trello (ClickUp, OpenProject)	allows the assignment and management of tasks (e.g. deadlines, subtasks), their visualization on Kanban boards, project management using Gantt-like charts and the development of a roadmap for team collaboration	hesitancy of group members to add new tools to their workflow; free options have feature limitations and paid options can be cost prohibitive for large teams

^a^See footnote 3.

### 3.5. Considering open science

‘Open science' is the label given to a broad range of policies and practices that promote transparency and accessibility in science [45]. In our experience, open science ideals often align well with the goal of BTS initiatives. If one aims to leverage people power to accelerate science, creating openly sharable and re-usable products—such as materials, data and code—is yet another way to accomplish that goal.

Open science prescribes transparency and accessibility beyond the members of a BTS project. Pre-registering key hypotheses and hypothesis tests prevents later disagreements about the best way to analyse the data and can also help avoid the appearance (or reality) of HARKing (i.e. Hypothesizing After Results are Known) [46] and biassed data-dependent analyses (e.g. *p*-hacking) [47–49]. Having manuscripts accepted in-principle at journals that use Registered Reports helps potential collaborators better understand the expected benefits of the project—essentially eliminating the risk that comes with investing resources into a project that may yield difficult-to-publish results—and provides an opportunity for feedback from potentially sceptical reviewers at a time when it can be most impactful for the project [30].

There are additional practical reasons for engaging in open BTS. For example, controlling access to team-wide documents is easier if the materials are open and accessible from the start. The alternative is often a time-consuming process of onboarding, granting access to individual parts of the project and creating privacy and data-sharing agreements across institutions. Similarly, making these organizational documents openly available allows others to adapt the materials and to understand the collaboration context (e.g. when documenting the history of seminal BTS projects [50]). Of course, projects will vary in the extent to which they can *ethically* be made open. For example, a team collecting potentially sensitive data could make their materials but not raw data open and shareable. In our experience, it is best to consider these issues at the beginning of the project—especially before collaboration agreements and workflows are developed.

## 4. Executing the project

Now that your BTS project is organized, it is time to get on with the business of performing research. While each BTS project will vary, we give some guidance here on a few aspects of this process.

### 4.1. Ethical approvals

Some BTS projects require ethical approval at multiple institutions, which can be extremely time-consuming. A good initial step is to contact a representative from your own ethics review board for guidance on this process. While doing so, here are two challenges you might have to consider: (i) differences in ethics review procedures and (ii) site-dependent ethical considerations. Below, we provide a brief overview of some issues we have encountered. For more information, see [51,52].

#### 4.1.1. Differences in ethics review procedures

Often, BTS initiatives require that data-collecting collaborators secure their own ethics approval—often while providing materials that can help streamline the process. Despite the fact that collaborators will probably have to make adjustments to fit into their own ethics review process, copies of completed applications, example consent forms and protocol descriptions are often extremely helpful and appreciated. Providing copies of ethics review certificates from other institutions is also sometimes beneficial, as some ethics boards may be willing to rely on pre-existing approvals when evaluating the project.

One of the biggest challenges in obtaining ethical approval for BTS initiatives is navigating variability in ethics review procedures across institutions and countries. Specifically, some institutions/countries do not provide ethics oversight for certain forms of research, whereas others have rigorous and structured requirements. Some ethics boards require processing fees (sometimes over USD $1000), whereas others are free. Some ethics boards take days or weeks to approve an application, whereas others may take many months. Furthermore, the forms and materials needed for ethics review vary from place to place.

When navigating differences in procedures, many have found it useful to both (i) establish minimum standards and (ii) allow contributors to move beyond those standards as required by their institution/country. For example, although not all institutions will require formal ethics review, you might require collaborators to commit to an existing standard (e.g. the Declaration of Helsinki [53]) or sign a project-specific document outlining ethical expectations (e.g. informed consent and confidentiality). It is also helpful to consider the extent to which your standards could become a barrier to participation. For example, requiring each site to receive independent approval from its ethics review board—perhaps with high application processing fees—might negatively impact the geographical diversity of your initiative. In these contexts, it is useful to consider (i) whether regional rules require review from an ethics board, (ii) if not, whether the project leads want independent ethics review to be a minimum standard at all research sites, (iii) whether ethics review reliance agreements are possible, and (iv) whether project funds can be used to offset ethics application processing fees.

#### 4.1.2. Site-dependent privacy considerations

Particularly in international collaborations, BTS leaders will have to navigate a variety of site-specific privacy and data-sharing considerations. For example, the General Data Protection Regulation law enacted by the European Union in 2018 introduced relatively strict rules for collecting, storing and sharing personal data [54]. Notably, organizations anywhere are required to follow these rules if they collect or target data related to individuals in the EU. Similarly, variation in regional laws means that the type of questions that are considered identifiable and/or sensitive varies across research sites. Relatedly, questions that seem benign in some world regions—such as ones concerning sexual orientation—are extremely sensitive in world regions where, for example, homosexuality is punishable by imprisonment or even death.

Collecting sensitive data across different sites is feasible, but often requires legal expertise and guidance (e.g. to complete Standard Contractual Clauses (SCCs) for the export of sensitive data outside the European Union) [55]. To avoid these challenges, many BTS initiatives opt to make the collection of sensitive data optional across sites unless it is absolutely necessary for the research question of interest. For example, most *ManyBabies* projects ask contributing laboratories to handle any necessary video coding locally before sharing de-identified tabular data with the central analysis team. Similarly, these projects encourage, but do not require, collaborators to share video recordings of their participants' testing sessions, which can be useful for quality control and secondary analyses. Projects that do require video sharing for analysis purposes have done so using SCCs between institutions to ensure appropriate data protection.

### 4.2. Data collection

When data are collected at more than one site, researchers must make choices about the extent to which aspects of the data collection protocol—such as surveys, stimuli, equipment and apparatus—are standardized versus allowed to vary. For example, many studies run by the *Psychological Science Accelerator* are highly standardized, using the same shared website (e.g. Qualtrics) to present, collect and store data. Similarly, the *Strengthening Democracy Challenge* provided a highly standardized test of 25 interventions by using the same measures and sampled population—which made the effects of the different interventions highly comparable.

There are a few reasons why BTS initiatives may opt for non-standardized data collection. First, high levels of standardization are not always possible. For example, developmental psychologists who participated in the first *ManyBabies* project expressed a need to retain as much of their usual set-up as possible to avoid disrupting their other ongoing studies. In addition, there was considerable variation in the equipment laboratories used to present stimuli to infants, which made a single software implementation impossible.

High degrees of methodological standardization may also be undesirable. Using the same set-up across sites bears the risk that the obtained results are specific to this particular set-up. By contrast, consistent results across variations in the set-up which are not thought to be related to the measure of interest may strengthen one's confidence in the robustness of the finding. Conversely, variation in the results due to varying features of the set-up may help identify important moderators. Sometimes methodological variation is also *desirable*, such as when a particular methodological feature is inappropriate or difficult-to-interpret in a certain world region. Regardless of why methodological variation occurs, we have found it extremely useful to document where and how it occurred. For example, the first *Psychological Science Accelerator* project asked laboratories to videotape their data collection procedure in case it might be useful for later secondary analyses or training purposes [9].

Ultimately, decisions about standardization and variability will be specific to the research question and goals of the project (e.g. is it meant to be a strict replication of an existing finding, or a test of the generalizability of a robust or controversial phenomenon?). For a detailed discussion of decisions related to centralized standardization versus allowing variation across individual laboratories in the first *ManyBabies* project, see [32]. For focused guides on big team data collection, we also recommend [56,57].

### 4.3. Data management

Standardization is also a central concern for data management in BTS. The lack of a standardization plan can create misunderstandings, additional work for standardization later on and potential for data loss due to unusability. Standardization can be achieved by several simple principles; variables should have similar names, values should have similar meanings and files should have similar structures. For example, although the key looking time measure in one data template for the first *ManyBabies* project was defined in seconds, many laboratories reported looking time in milliseconds. Similar mismatches occurred in a number of fields across the 69 datasets the analysis team received [32]. The process of validating the data by hand was so time-consuming that *ManyBabies* has since developed a data validator for laboratories to verify their data files are properly formatted before uploading to project servers (https://manybabies.shinyapps.io/validator/) [58].

### 4.4. Data analysis

Often, a central challenge for a BTS project is agreeing on an analysis plan. In BTS, there can be an unusually large number of people looking at an analysis plan, working on data processing and analysis, and reviewing accompanying code. This comes with many benefits—but also occasional challenges. For example, when working with a large team, it is not unusual for collaborators to have different ideas about how to process and analyse the data. Later, we discuss ways that these disagreements (and others) can be handled. For now, though, we focus on the logistics of leveraging BTS for data analysis.

Several BTS initiatives leverage the power of collaboration when analysing data. For example, complicated data processing and analysis workflows can be broken up and assigned to different collaborators. Collaborators can also review code to check that it can be independently reproduced and understood by others. Some initiatives have also had a large team of collaborators independently develop and execute their own data analysis plans. This strategy has helped to highlight how justifiable differences in data processing and analysis strategies impact scientific conclusions [19]. For more guidance on these ‘many analysts' projects, see [59].

When working with a team on data processing and analysis, well-documented code, version control and good package management are absolutely essential. After all, if multiple collaborators are editing data processing scripts, there is an increased risk that they will inadvertently overwrite each other's work. Tools such as Git and GitHub can facilitate code sharing and version control, while the use of package management software can ensure that numerical results do not change due to small changes in the software stack. These tools allow for transparent and reproducible workflows for both contributors and consumers of BTS research.

## 5. Completing the project

Once you have collected and analysed data, how should you disseminate your findings and products? Most BTS projects will result in a publication in a scientific journal. However, the publication process often takes a long time and requires stewardship to establish and maintain momentum.

We recommend that the leadership team sends progress updates to the larger team when milestones like finishing data collection or data analysis are met. Conference presentations can also be a great way to update the larger research community on the status of large projects. For example, the results of the *Strengthening Democracy Challenge* were emailed directly to the developers of the interventions, presented at a conference organized by the leadership team and disseminated to the public in a Twitter thread, a press release and a publicly available working paper [16].

### 5.1. Authorship

Authorship remains one of the privileged avenues of recognition in academia and influences a number of important professional outcomes, including job placement, promotion and funding of future research. Yet, many researchers have pointed out that our current authorship models do not sufficiently support BTS collaborative research (e.g. how should inclusion among dozens if not hundreds of co-authors be valued?) and have proposed an alternative contributorship model [60,61]. Such authorship reform is promising, but slow and ongoing [62]. Thus, many BTS researchers will find themselves balancing their need to survive in the current system with their desire to contribute to reform.

One of the biggest decisions that many BTS initiatives grapple with from the onset is determining who is eligible for authorship. Although we advocate for maximally inclusive authorship policies to ensure that all substantive contributions are credited, the rules governing this issue vary from journal to journal and from field to field. For example, the International Committee of Medical Journal Editors requires that all authors make a substantial contribution to writing, but many journals do not (e.g. *Nature*) [63]. These policies often end up being a key consideration when it comes to determining where to publish BTS manuscripts.

Another big decision is whether to (i) list individual authors or (ii) publish under a consortium name (e.g. the Open Science Collaboration [20]). Consortium names are a great way to highlight the collective nature of BTS and avoid the hassles of compiling large authorship lists [13]. However, we have found that many researchers—particularly those from under-represented world regions—do not benefit much from papers with consortium authorship. Some researchers have indicated that papers simply do not count for them in hiring, promotion or funding if their name is not listed on the author by-line. Although the members of the consortium can hypothetically be listed in the article metadata, we have found that many journals do not do so by default. Relatedly, it can be difficult for search engines and reference databases to correctly attribute individual contributions to a paper that lists a consortium as the sole author. Thus, many of our collaborations have moved away from consortium authorship and towards more traditional—but extremely long—lists of individual authors (e.g. [9]). To aid this process, many of us have found it useful to (i) first check what information the journal submission portal requires, (ii) have authors add this information to a spreadsheet (e.g. a Google Sheet) or form (e.g. Google Form), and then (iii) use the *tenzing* Web-based app and R package application to compile information about affiliations, funding and contributions [64,65]. We have also found it useful to discuss the long list of authors ahead of time with journals; given that many journal submission systems are not well-equipped to handle large lists of co-authors, several editors have granted exceptions or amendments to usual submission procedures.

Because decisions about authorship are so consequential, we strongly recommend identifying a target journal, authorship model and contributor tracking strategy *before* beginning the collaboration (or as early in the collaboration as possible) and documenting these decisions in a collaboration agreement. In the absence of such planning, you will need to retroactively work towards a unanimous decision about authorship. After all, BTS outputs are often shared products, so everybody needs to agree about the rules governing attribution for that shared product. To facilitate this process, you may find it helpful to poll collaborators and host shared discussions about next steps.

### 5.2. The writing process

When it is time to write, the distributed nature of BTS can be both a help and a hindrance. At its best, the process of co-writing with a large group can be exhilarating. One of us remembers the thrill of working on the Open Science Collaboration paper [20], where it seemed like all 270 authors were simultaneously working on the same Google Doc, debating what it meant to have failed to replicate an empirical finding. At its worst, though, collaborative writing can lead to unstructured documents, endless lists of comments and edit wars. Here are a few ideas that have helped us move forward in shared writing projects, borrowed from both our experience and recommendations by Moshontz *et al*. [29].

We have found it useful to have a small group, often composed of project leaders, write a first draft—or at least a comprehensive outline. Starting from a blank page can be daunting, and co-authors may not know (or have different ideas about) how to frame the paper. Having a first draft prepared allows collaborators to focus on evaluating the structure, refining ideas and writing and commenting on big-picture issues.

Once collaborators are invited to jump into the draft, we find it useful to encourage them to write, rather than merely comment. ‘Track changes by default’ is our mantra. It can be overwhelming as a team leader to come back to a manuscript where your collaborators have marked each and every sentence with comments. But if a co-author directly edits text to point out a problem and provide a solution, it is much easier to judge and respond. Contributors new to the process (especially more junior contributors) may need explicit permission to feel empowered to dig into the document and edit the work of others. Comments should be used sparingly in cases where you have identified a problem but are not able to fix it yourself or where you feel that further active discussion is needed due to a difference of opinion.

Just as it is useful to have a dedicated team write the first draft, we have found it useful to have a dedicated team (or person) periodically clean up the draft. When dozens of collaborators contribute to writing, the resulting manuscript often lacks coherence until one person goes through to connect ideas and refine the prose. It is helpful to give collaborators deadlines for writing and commenting on a draft. The author who is then leading the draft can work to resolve comments, accept or reject changes and (if disagreements persist) create a narrowed list of issues to discuss at a future meeting.

Of course, there are many different ways that large teams can navigate the writing process. Some smaller BTS initiatives have collaborators edit their own copy of the manuscript, which are then either merged into a single file or reviewed one at a time. For a particularly large project run by the *Psychological Science Accelerator*, they asked their 450+ collaborators to submit feedback through a structured survey form rather than directly editing the manuscript [66]. This form, among other things, asked co-authors to distinguish between major feedback (e.g. disagreement with a main conclusion) and minor feedback (e.g. disagreement with the use of an Oxford comma). An added benefit of this approach is that all feedback could be exported to a spreadsheet, which allowed the lead team to organize the feedback and their subsequent responses.

### 5.3. Contribution tracking

As the size of a BTS initiative grows, it becomes impractical for project leaders to keep track of the myriad ways individuals contribute to the project. Consequently, many BTS initiatives establish ways for collaborators to self-report their contributions—ideally specifying this arrangement alongside other information about authorship expectations in a collaboration agreement. For example, one way to do this is to have a shared spreadsheet or a survey/form which contributors can update throughout the project to keep a record of the ways in which they have contributed. To organize these reports, many of our groups have opted to use a standardized taxonomy, like the Contributor Roles Taxonomy [67], which allows collaborators to report contributions for up to 14 categories (e.g. conceptualization, analysis, writing). Many also opt to report these self-reported contributions in the manuscript so that readers can understand who did what on any given project.

## 6. Navigating disagreements

Many issues that we have discussed—such as open science and collaboration agreements—are relevant to *all* stages of a BTS project. Another one that we feel is particularly important is navigating disagreements.

As the number of co-authors increases, so does the likelihood of encountering disagreements. Disagreements should be expected, and they are often useful! They can lead collaborators to refine their thinking, improve their methods and form more sound conclusions. Unfortunately, navigating disagreements can also introduce delays and stress—and some disagreements may be irreconcilable. In a field where papers and citations are currency, we believe that researchers should not lose their right to receive formal recognition for their contributions to a scientific endeavour just because they do not agree with all the conclusions in the paper [62]. So how should we navigate such disagreements?

There are four general approaches that we have seen thus far. Most simply, you can continue to work on establishing consensus, perhaps by setting up meetings with dissenting parties and a mediator who helps them productively discuss their disagreements. Another approach we have successfully deployed is decision by a neutral arbiter. The disagreeing parties agree on a neutral arbiter, state and debate their cases in the presence of the arbiter and agree *a priori* to let the neutral arbiter make the final decision. A third approach, developed in the *Many Smiles Collaboration* [8], is to deploy a majority–minority opinion model. In this model, the main text of the paper reflects the majority opinion among collaborators, and special sections (e.g. electronic supplementary material) are developed where dissenting opinions are described. Ultimately, the *Many Smiles Collaboration* was completed with no major outstanding dissenting opinions; anecdotally, though, the option to upload a dissenting opinion diffused many contentious discussions. A fourth approach, used by the *Many Analysts Religion Project* [68], involved coordinating a special issue that contained both a main article and commentaries submitted by co-authors. Such an approach gives co-authors the opportunity to communicate ideas—including, but not limited to, dissenting opinions—that otherwise would not make it into the main article.

Whatever approach you decide on, it is useful to plan for disagreements early on in the project. For example, one may include a brief description of how disagreements will be handled in the project's collaboration agreement. Similarly, disagreements about data analysis procedures—how to handle outliers, which mathematical models to use, which covariates to include in models—can be addressed before data collection commences by developing a pre-registered analysis plan.

## 7. Conclusion: the future of big team science

If you have made it this far, we hope you are still as enthusiastic about BTS as we are. The advice we have presented is intended to capture (i) what we wish we had known when we started our own initiatives and (ii) lessons we have learned along the way. We hope you take what you can use and leave the rest, depending on the nature and scope of your project.

Over the last 5–10 years, behavioural scientists have increasingly explored BTS research, largely using a trial-and-error approach. In the next 5–10 years, we anticipate that the field will transition to a more mature style of BTS, as our understanding of best practices across the diverse BTS networks improves. In fact, the multi-network group we formed to share and learn from each other—the Big Team Science Lab (bigteamsciencelab.github.io)—was where the idea for this ‘How-To Guide' was born. During this transitional period, it will be important to create and maintain the infrastructure necessary to support this new approach to behavioural science. New tools to support the collection, storage, processing and analysis of experimental data are needed, as is training a next generation of researchers with the necessary skills to lead and participate in such initiatives. We hope that this guide helps to disseminate some of our ‘lessons learned' from prior BTS projects.

We also hope that this guide elucidates some of the important benefits of BTS. First, BTS projects can provide a pipeline for training and mentoring new sets of researchers. Many BTS initiatives effectively create a network of scientists, allowing new researchers to get involved with impactful projects more easily, even if they do not already have connections to an established laboratory. Second, the large scale of BTS requires researchers to put more effort into tasks that ensure smooth and effective communication among collaborators, such as developing standards for data wrangling, analysing and visualizing the data, commenting and proofreading code, writing better accompanying documentation and encoding metadata. These features make BTS projects well equipped to train researchers in open science practices (e.g. open data, open laboratory/notebooks, open-source software), which is vital due to the growing number of open science mandates implemented by funding agencies worldwide [69]. Third, by decentralizing the processes of research design, stimulus creation, data collection and data analysis, BTS projects can make high-quality research more accessible for people working in laboratories lacking resources. Fourth, BTS projects increase epistemic diversity. For example, tournaments that test many ideas at the same time often recruit submissions of researchers from different disciplines or from outside of academia. Such collaborations introduce researchers to new ideas from their colleagues and may improve the dialogues between researchers and practitioners. Taken together, these benefits mean that large teams can make science more open and inclusive, allowing more scientists to contribute ideas and increasing the potential for novel discoveries.

Although there are still difficulties to overcome, we feel that BTS is a powerful tool for creating robust and generalizable knowledge. Although our focus has been on the behavioural sciences, we suspect that many of our lessons learned will generalize to other disciplines. We hope you are inspired to adapt the BTS approach to your topics and questions, and that the information we have provided here will help you get started. We also invite researchers to contact us directly if further guidance would be of benefit. After all, we have benefited from such guidance when establishing our own BTS initiatives. The biggest challenges facing our world require the power of many minds, and this power can be leveraged not only in the implementation—but also the planning—of BTS initiatives.

## Data Availability

Author contributorship information and coding used to produce figure 1 are available at: https://osf.io/2p4ct/. The data are provided in electronic supplementary material [70].

## Footnotes

1 A precise definition of ‘big team science’ has proved elusive, so we use a relatively inclusive definition in this paper: collaborations involving a coordinated effort from an unusually large number of researchers. However, there are several ambiguous cases that we do not discuss in the manuscript, including scenarios where a large number of researchers are considered *participants* in a study.

2 We broadly define diversity as variability in any given domain (or combination of domains). For example, this includes variability in theoretical perspectives, lived experiences, career stages, cultural backgrounds and more [2].

3 This list of tools is intended to provide guidance on the types of tools that we have found helpful and their characteristics. In some cases, geographical availability varies (e.g. all cloud software products are not available in all regions of the world). Furthermore, it is likely that the best option will change with time as new platforms and applications become available and replace older systems.
